# Evaluation of Computer-aided Strategies for Teaching Medical Students Prenatal Ultrasound Diagnostic Skills

**DOI:** 10.3885/meo.2008.Res00275

**Published:** 2008-09-24

**Authors:** Lawrence S. Amesse, Ealena Callendar, Teresa Pfaff-Amesse, Janice Duke, William N.P. Herbert

**Affiliations:** * Division of Reproductive Endocrinology and Infertility, Department of Obstetrics and Gynecology, Wright State University Boonshoft School of Medicine, Dayton, Ohio, USA; † Department of Obstetrics and Gynecology, University of Virginia School of Medicine, Charlottesville, Virginia, USA

**Keywords:** Computer-based learning, prenatal ultrasound, diagnostic skills, 3^rd^-year medical students

## Abstract

**Objective:**

To evaluate whether computer-based learning (CBL) improves newly acquired knowledge and is an effective strategy for teaching prenatal ultrasound diagnostic skills to third-year medical students when compared with instruction by traditional paper-based methods (PBM).

**Study Design:**

We conducted a randomized, prospective study involving volunteer junior (3^rd^ year) medical students consecutively rotating through the Obstetrics and Gynecology clerkship during six months of the 2005–2006 academic year. The students were randomly assigned to permuted blocks and divided into two groups. Half of the participants received instruction in prenatal ultrasound diagnostics using an interactive CBL program; the other half received instruction using equivalent material by the traditional PBM. Outcomes were evaluated by comparing changes in pre-tutorial and post instruction examination scores.

**Results:**

All 36 potential participants (100%) completed the study curriculum. Students were divided equally between the CBL (n = 18) and PBM (n = 18) groups. Pre-tutorial exam scores (mean±s.d.) were 44%±11.1% for the CBL group and 44%±10.8% for the PBL cohort, indicating no statistically significant differences (*p*>0.05) between the two groups. After instruction, post-tutorial exam scores (mean±s.d.) were increased from the pre-tutorial scores, 74%±11% and 67%±12%, for students in the CBL and the PBM groups, respectively. The improvement in post-tutorial exam scores from the pre-test scores was considered significant (*p*<0.05). When post-test scores for the tutorial groups were compared, the CBL subjects achieved a score that was, on average, 7 percentage points higher than their PBM counterparts, a statistically significant difference (*p < *0.05).

**Conclusion:**

Instruction by either CBL or PBM strategies is associated with improvements in newly acquired knowledge as reflected by increased post-tutorial examination scores. Students that received CBL had significantlyhigher post-tutorial exam scores than those in the PBM group, indicating that CBL is an effective instruction strategy in this setting.

A computer-based learning (CBL) approach to student teaching is an emerging field of instruction that holds great promise in contemporary medical education. Some educators believe that interactive computer programs can enhance and may, in some cases, replace traditional lecture-based formats.^1^,^2^,^3^ One unique aspect of CBL is that it has the capability of producing and guiding self-assessment exercises in private. Students with differing abilities and/or levels of training can access individual instruction paths that allow them to learn the same comprehensive educational material.^1^ This contrasts sharply with more static learning modalities such as attending formal courses, lecture-based learning or some paper-based methods. Computer-aided instruction can be incorporated into a variety of learning environments. It provides the learner with a variety of images, sounds and actions that render the learning process an interactive event irrespective of the learner's location. Additionally, a large number of students can receive instruction with limited expenditure of faculty time. Once developed, CBL programs offer a flexible and extremely assessable way of presenting large amounts of information through web-based learning; they encourage personal exploration of an unlimited amount of detailed knowledge often required for medical education.^1^,^3^

Although many interactive computer-assisted programs have been described, few prospective, randomized, controlled studies have definitively evaluated in medical education the effectiveness of this approach over standard, paper-based learning.^2^,^4^,^5^ In a recent analysis of 12 studies conducted in resident and medical student populations in which CBL was compared with traditional format-matched methods, only five (42%) reported significant improvements in learning by computer-based strategies.^5^ Devitt and coworkers demonstrated improved student learning in ophthalmology using computer-aided instruction.^6^ This was supported by Hallgren and colleagues who reported that web-based tools with self-evaluation exercises were effective in improving student test scores on anatomic structure identification exams.^7^ In contrast, Khalil et al. observed that interactive computer-assisted learning programs, when compared with paper-based methods, showed no significant differences in immediate recall of anatomic structures. However, the Khalil group identified important differences in the attitudes of the participants. Specifically, a significant number of students perceived computer-based imagery as a better strategy for assimilating information.^8^ Other studies have shown increased satisfaction on the part of the learner for CBL, suggesting that this type of teaching is well received by participants.^5^,^8^–^11^

That some studies have demonstrated comparable outcomes for CBL and PBM may be a reflection of the quality of the computer program and/or the subject matter being taught.^7^,^8^ Indeed, it is possible that instruction by CBL may be better suited for certain teaching formats. This may also account for the variation in study outcomes.^11^,^12^ One format that appears to favor CBL is an approach involving visual imagery.^13^,^14^ Training in obstetrics and gynecology requires extensive visualization and spatial learning. Moreover, digital and imaging components used in performing ultrasound are well suited for interactive CBL programs.^5^ To this end, prenatal ultrasound imaging may represent a candidate learning format to test the overall effectiveness of CBL. In this study, we sought to evaluate in third-year medical students whether CBL improved newly acquired knowledge and was an effective learning strategy for teaching ultrasound prenatal diagnostic skills.

## Materials and Methods

The institutional review board at the Miami Valley Hospital approved this study. Between September 2005 and February 2006, a total of 36 third-year medical students (19 females, 17 males) consecutively rotating through the Obstetrics and Gynecology clerkship at Miami Valley Hospital volunteered to participate in this study. All subjects were matriculates of the Wright State University Boonshoft School of Medicine in Dayton, Ohio, and had completed that school's Biennium I core curriculum as well as passed step I of the USMLE licensing examination. Although they had been exposed to basic concepts of genetics during pre-clinical years, all students had limited exposure to obstetrical ultrasound prior to taking part in the study. Participants all owned and regularly used computers, and had experience in computer testing through the USMLE examination. Some had used interactive computer programs in the past.

The curriculum was composed of three parts: the pretest, the CBL or PBM tutorial session, and the post-instruction examination. The testing and learning sessions were carried out during designated periods of time in the obstetrical portion of the rotation. There was no definitive time limit for task completion. The entire curriculum took an average of 90 minutes per student to complete. The students were not required to complete the entire program at one time. All students were provided with a brief introduction to the testing and tutorial formats and asked to “do their best.” All results from the study were confidential. The students were informed that examination results would not be used for grades or evaluation purposes and that they would not be provided with the examination results. No extra credit was awarded for participating in the study.

**Figure 1:**
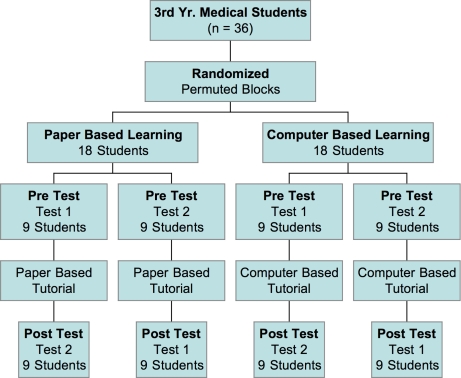
Flow of participants and study design.

The study, shown schematically in Figure 1, was based on pretest and posttest group design. Thirty-six participants were randomly assigned to permuted blocks with half assigned to the CBL group (n = 18) and the other half (n = 18), to the PBM group. Two equivalent examinations, “Test 1” and “Test 2,” were developed. Within their respective groups, the participants were further randomized to take either “Test 1” as the pre-test and “Test 2” as the post-instruction exam or the reciprocal test set, such that all students were examined on the identical 64 questions, albeit in a different order. The subject content and selected images used in the test questions consisted of 34 fill-in-the-blank style questions. The questions assessed the participants’ newly acquired knowledge of major congenital abnormalities identifiable on prenatal ultrasound imaging along with associated chromosome anomalies. Questions on both tests contained both CBL and PBM components. Test 1 was composed of 22 computer-posed questions and 12 paper-written questions, whereas Test 2 was composed of the reciprocal format. An example question would present an ultrasound image of a fetus with a cystic hygroma and require the student to identify the anatomic anomaly along with the associated chromosomal abnormality, Trisomy 21.

Two obstetrics and gynecology educators (LSA and EC) organized and wrote the subject content for all three curriculum components. Near-identical instructional material, summarized in Table I, was represented in both tutorial formats. An IBM ThinkPad laptop computer was provided to each student. The “computer-based” tutorial was adapted from the interactive CD-ROM, “The Ultrasound Simulator,” by Dr. Lee.^15^ It was composed of real-time video segments as well as audio and interactive components.^15^,^16^ The CD-ROM was designed as a learning tool for practicing physicians and is used by obstetric and gynecology residents for training in prenatal ultrasound diagnostics. The following material was covered by the CD-ROM: (1.) ACOG Technical Bulletin 187, Ultrasound in Pregnancy; (2.) Research Library: key concepts, epidemiology and associated fetal ultrasound findings for Trisomy 13, Trisomy 18 and Trisomy 21 were presented in real time; (3.) self-assessment, which sought responses in relation to ultrasound findings.^5^,^16^,^17^

**Table 1.** Summary of instructional material for both tutorial programs.

Computer-Based Learning *Using CD-ROM*	Paper-Based Method*Using Program Notebook*
1. Review ACOG Technical Bulietin 187, “Ultrasound in Pregnancy” on Lee's CD-ROM, “Ultrasound Simulator”	1. Review ACOG Technical Bulietin 187, “Ultrasound in Pregnancy” Paper-Based (complete photocopy)
2. *Research Library:*	2. *Research Library:*
a. Review key concepts, epidemiology, 1st, 2nd & 3rd trimester U/S exams, discussion, prognosis, associated chromsome abnormalities for:	a. Review key concepts, epidemiology 1st, 2nd & 3rd trimester U/S exams, discussion, prognosis, associated chromosome abnormalities for:
Trisomy 13	Trisomy 13
Trisomy 18	Trisomy 18
Trisomy 21	Trisomy 21
b. Review ultrasound real-time computer images for: Trisomy 13, Trisomy 18, Trisomy 21	b. Review ultrasound photocopy images for Trisomy 13, Trisomy 18, Trisomy Trisomy 21

“Paper-based” was defined in this study as printed, structured text plus, high-quality black and white still photocopies of ultrasound images. Printed materials for the paper-based (control) group's tutorial contained near-identical instructional material to that described previously for the CD-ROM and included the following: (1.) Complete black and white photocopy of the ACOG Technical Bulletin 187, Ultrasound in Pregnancy, with supplemental material equivalent to pop-ups on the CD-ROM; (2.) Paper Based Research Library: key concepts, epidemiology and associated fetal ultrasound findings for Trisomy 13, Trisomy 18 and Trisomy 21 were presented in standard text on paper and photocopy still images.^15^–^17^

The program was considered completed when the participant finished all three components of the curriculum. One person (LSA) graded the examinations. The grader was blinded to the tutorial format as well as to the identity of individual participants. One point was given for each correct test answer, and no points were given for an incorrect answer. Partial credit was not given and no points deducted for incorrect answers.

## Statistical Analysis

In this pretest-posttest comparison group design study, changes in acquired knowledge were assessed by comparing differences in pre-test and post-instruction examination scores. Scores from the exams were expressed as mean percentages and standard deviations of correct answers. Test scores from the two pre-tutorial exam groups, Test 1 and Test 2, as well as from both CBL and PBM groups’ post-tutorial exams were analyzed parametrically on Graph Pad^®^ (GraphPad Software, Inc., San Diego, CA) using the two tailed student's t-test.

## Results

All 36 students completed the curriculum with half (18 students) taking “Test 1” as the pre-tutorial examination and the other half, “Test 2.” A perfect score for both examinations was 100% or 34/34 correct answers. No student achieved a perfect score for either exam. For the students taking pre-test “Test 1,” the mean (±s.d.) examination score was 44% ±9.2% and was similar to the 43%±12% mean (±s.d.) score achieved by students pre-test “Test 2.” The differences between the two pre-test groups were not statistically significant (p = 0.885), supporting the equivalency of the two test forms.

**Figure 2:**
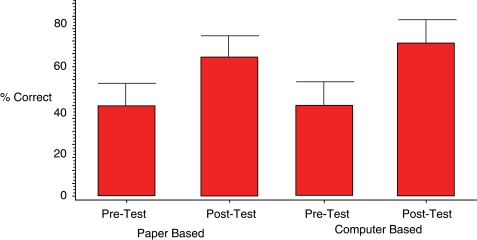
Pre-tutorial and post-tutorial examination scores for the paper-based and the computer-assisted learning groups.

Scores from both the pre-tutorial and post-instruction examinations for both instructional groups are summarized in Figure 2. The mean (±s.d) post-instruction score for students in the PBM arm of the study was 67% (±12%), representing an increase of 23 percentage points, on average, over the pre-tutorial exam score of 44% (±10.8%); this improvement was statistically significant (p < 0.0001). Similarly, the mean (±s.d) post-tutorial test score for CBL students increased from 44% (±11.1%) to 74% (± 11%). This represented an increase of 30 percentage points, a statistically significant improvement (p <0.0001).

**Figure 3:**
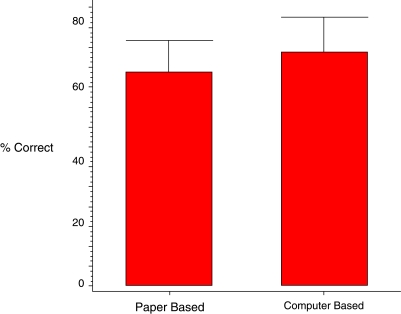
Post-tutorial examination scores for both paper-based and computer-based groups.

Post-tutorial examination scores from both the CBL and the PBM groups were compared. These data are summarized in Figure 3. The post-test score (mean±SD) for the CBL group was 74% (±11%) and 67% (±12%) for the PBM group, with the CBL group achieving an average exam score 7 percentage points higher than that of the PBM group. The difference in the scores was statistically significantly (p = 0.0488). The results indicate that both instruction groups achieved significant increases in post-training exam scores, with subjects in the CBL group achieving higher scores than traditionally-instructed PBM students.

## Discussion

This prospective, randomized study indicates that tutorial sessions by computer-assisted and paper-based approaches are associated with significant increases in newly-acquired knowledge as measured by test score improvements. This finding is consistent with results from similar, comparative studies.^2^,^8^,^11^ What is noteworthy is that our study revealed a significant difference in test performance between the two groups favoring instruction by the computer-assisted method. Specifically, students tutored by the interactive computer-aided approach had post-tutorial mean exam scores 7 percentage points (74% vs. 67%) higher than their PBL cohorts. Together, these data indicate that interactive computer-based learning represented an effective learning strategy.

In medical education, this report is among the first prospective, randomized studies that compares in the specialty of obstetrics and gynecology the effectiveness of computer-assisted learning with paper-based instruction. Indeed, in a recent descriptive study by Adler and Johnson, the authors reviewed over 1,000 articles published between 1966 and 1998 relative to computer-based instruction and reported that 60% of the articles were demonstration papers.^4^ Letterie, a reproductive endocrinologist, reported similar findings in his 2003 study.^5^ He also recognized that in obstetrics and gynecology there were no comparative studies that demonstrated advantages of computer-aided strategies over paper-based learning.^5^

The major critique of the published comparative studies has been that the tutorial material often differs between the two media, thereby confounding any meaningful comparison.^4^,^18^ In this study, we maintained a high degree of content fidelity to minimize such confounders. We chose an area of study, prenatal ultrasound diagnostics, which represented the first formal exposure of the subject to the students and had relevance to their training. The CBL format was not adapted to a web-based program, although theoretically it easily could have been, and there were no hyperlinks. Near-identical tutorial material was provided to both groups. The major differences in the instructional content were related to properties unique to the computer, such as video, audio and interactive real-time components, features impossible to replicate in traditional paper-based formats. These types of differences will always exist and, while some experts may view them as confounders of comparative studies, there is no way to reconcile the differences because of the inherent sophistication and technological advancements of computer-based systems as compared with paper. The best investigators can do is aim for well-designed, randomized, prospective studies that minimize confounders, after the manner of this study.

Diagnostic ultrasound is a visual and spatial medium. Both still and real-time imaging capabilities easily render ultrasound applications to a variety of computer formats and, by extension, ultrasound represents an ideally-suited medium for evaluating computer-based learning.^6^ Indeed, real-time imaging reinforces visual recognition and memory, enhances recall, and can facilitate learner achievement.^8^,^9^,^13^ This reinforcement is not possible with paper-based instruction, which may account for the significant differences identified in this study between the two tutorial groups.

Of note was that both pre-tutorial and post-instruction examination scores were relatively low. This may have been attributed to the testing format that used fill-in-the-blank type questions rather than multiple-choice questions usually represented in medical school examinations. Another consideration is that the tutorial content represented new material, so the repeated exposures often required for assimilation were lacking. Finally, while the Ultrasound Simulator is a valuable learning program, it is not specifically designed for medical students. For this reason, only a portion of the program was used.

Our study's results indicate that interactive computer-based instruction was associated with improved learning when compared with a paper-based method, boding well for the continued development and use of this approach. Additional prospective, randomized studies involving larger groups of participants will be necessary to confirm these findings. Computer- and web-based programs are expected to become an integral part of medical student curricula.^6^,^19^ There is a need for additional medical student computer programs in obstetrics and gynecology instruction. There is also a need for properly designed computer-aided formats specifically intended for this and future generations of medical student learners. These learners are computer-savvy with different learning styles from their predecessors.^20^ The exponential growth of basic medical knowledge and the diversity of new diagnostic aids require that more efficacious, all-encompassing, personal forms of learning be developed.^1^ Furthermore, it is important that they be systematically evaluated for their effectiveness before being implemented on a wide scale.
